# 

**Published:** 1893-03-04

**Authors:** 


### CONTENTS.

The Prevention of Consumption - - - - - - - 355

Around the Hospitals - - - - - - - - - 356

Medical History from the Earliest Times. By E. T. WITHINGTON, M.A., M.B. Oxon. XLV.—The Revival of Learning 357

Everybody's Page - - - - - - - - - - 358

Diet in Disease. By Mrs. ERNEST HART. XIV.—Diabetes (continued) - - - - - - - - - - - 359

Contemporary Theory and Research - - - - - 360

Annotations.—The Doctor Day by Day—Strength—Influenza Once More - - - - - - - - - - 361

Notes and News - - - - - - - - - - 362

The Institutional Workshop—

WITHIN THE HOSPITALS.—The Hull Royal Infirmary - - - 367

PRACTICAL DEPARTMENT.—Lockers - - - - - - 368

The Hospital Clinic—

PADDINGTON GREEN CHILDREN'S HOSPITAL.—Treatment of Rickets (concluded) - - - - - - - - - - 333

THE HOSPITAL FOR CONSUMPTION, BROMPTON.—The Treatment of Bronchitis - - - - - - - - - - - 364

ROYAL INFIRMARY, EDINBURGH.—Treatment of Nævus - - 365

THE PRACTITIONERS' BOOKSHELF.—"The Chemistry of Life and Health"—Progress of Hygiene - - - - - - - 366

NEW DRUGS, APPLIANCES, AND THINGS MEDICAL - - - 366

The Radcliffe Infirmary, Oxford - - - - - - 369

Construction Notes - - - - - - - - - 370

The Editor's Letter-Box.—Mr. Lawson Tait and Vivisection 370

Scraps and Gleanings - - - - - - - - - 370

The Student of Medicine.

### EXTRA SUPPLEMENT.—THE NURSING MIRROR.

News from the Nursing World.—Sick and Poor—Fixed v. Movable Feasts—Home Missions—Progress—A Cottage Hospital — Practical Cyclists — Eccentric Instruction—Nursing in Paris—Nursing for Natives—Short Items—The Disabled Nurse—"The Hospital" Endowed Bed - - clxv

Management of Consumption. IV.—Coughing - - clxvii

Parochial Nursing in Glasgow - - - - - clxvii

The London Hospital, Whitechapel - - - - - clxviii

Everybody's Opinion.—Nurses' Holidays—Dying: not Drunken—Carbolised Sheets—"The Hospital" Endowed Bed clxix

Cholera and Common Sense.—The Nurse Dolls - clxix

The History of Nursing.—In Memoriam - - - clxx

Notes and Queries—Wants and Workers - - - clxxi

For Reading to the Sick.—Burdens - - - - - - clxxi

Some Australian Experiences.—My Resignation - - clxxii

The Book World for Women and Nurses - - - clxxiii

